# Lifestyle and behavioral modifications made by patients with interstitial cystitis

**DOI:** 10.1038/s41598-021-82676-5

**Published:** 2021-02-04

**Authors:** Krystal Bay-San Lin, Ming-Ping Wu, Yen-Kuang Lin, Yu-Chun Yen, Yao-Chi Chuang, Hung-Yen Chin

**Affiliations:** 1 Department of Obstetrics and Gynecology, Taipei Medical University Hospital, School of Medicine, College of Medicine, Taipei Medical University, Taipei, Taiwan; 2 Division of Urogynecology, Department of Obstetrics and Gynecology, Chi Mei Medical Center, Tainan, Taiwan; 3 Department of Obstetrics and Gynecology, College of Medicine, Fu-Jen Catholic University, New Taipei City, Taiwan; 4 Biostatistics Center, Taipei Medical University, Taipei, Taiwan; 5 Biostatistics Center, Office of Data Science, Taipei Medical University, Taipei, Taiwan, Republic of China; 6 Department of Urology, Kaohsiung Chang Gung Memorial Hospital, Chang Gung University College of Medicine, Kaohsiung, Taiwan; 7 Department of Obstetrics and Gynecology, Taipei Medical University Hospital and College of Medicine, Taipei Medical University, No.252, Wu Hsing Street, Taipei City, 110301 Taiwan, Republic of China

**Keywords:** Diseases, Health care, Urology, Urogenital diseases

## Abstract

Interstitial cystitis/bladder pain syndrome (IC/BPS) negatively affects the quality of life. In this study, we investigated the lifestyle behavioral changes patients with IC/BPS make to cope with their symptoms. This prospective study was conducted between August 2018 and June 2019. All patients had a primary symptom of suprapubic pain with a full bladder and other lower urinary tract symptoms for more than 6 weeks as well as cystoscopic findings. All participants completed our self-developed questionnaire, which included informations about their living and work environment, occupational garments, dietary habits, and personal habits. Continuous variables were compared using an independent sample *t* test, and categorical variables were compared using a chi-square test. We recruited 86 patients with IC/BPS and age-matched 86 controls without IC/BPS. In our study, patients with IC/BPS had more cranberry intake (45.34% vs. 5.81%, *P* < 0.05) than non-IC/BPS controls; the IC/BPS group had decreased consumption of coffee and spicy food; and wore less makeup or special work garments. In conclusion, patients with IC/BPS tend to make several lifestyle behavioral changes to cope with their symptoms.

## Introduction

Interstitial cystitis (IC) or interstitial cystitis/ bladder pain syndrome (IC/BPS) is a heterogeneous condition involving chronic and often severe pain in the urinary bladder^1^. According to the European Society for the Study of Interstitial Cystitis, IC/BPS is diagnosed on the basis of the presence of chronic pelvic pain or discomfort related to the bladder and at least one other lower urinary tract symptom (LUTS), such as urinary frequency or urgency^2^. Our previous study showed patients with LUTS are associated with subsequent increased risk of outpatient visits and hospitalizations^3^. In addition to the healthcare seeking behavioral changes, the threshold of visiting the emergency room decreases either due to medical reasons, but also due to mental dependency^3^. Our study on IC/BPS indicted the similar medical healthcare-seeking behavioral changes among them (manuscript in revision), and a significantly higher incidence rate of anxiety, depression, and insomnia^4^.

The etiology of IC/BPS remains poorly understood, but a number of pathogenic mechanisms have been proposed, including bladder epithelial dysfunction, epigenetic reprogramming of the bladder uroepithelium, autoimmune conditions, and nitric oxide synthase imbalance^5^. The complexity of IC/BPS is reflected by the multitude of etiologic factors implicated in the pathogenesis of the disease, absence of universally accepted disease definitions, and lack of appropriate tools to discriminate individuals having the actual disease from those with transient manifestations of bladder pain^5^. It has a negative impact on several aspects of life, for instance, quality of life and sexuality^6^. Hung et al. reported that patients with overactive bladder (OAB) tend to make some adaptations, such as locating restrooms in unfamiliar places, restricting fluid intake, avoiding outdoor activities, limiting attendance of social gatherings, making efforts to conceal odors and to maximize hygiene, using pads, and avoiding sexual intimacy^7^. Therefore, patients with IC/BPS may choose to make some lifestyle modifications to cope with their LUTS symptoms.

We were especially interested to see if the elements, including those regarding diet from Hung et al. and other environmental factors listed in Altman et al^5^, would cause motivate IC/BPS patients to choose to relocate, change workplace, or lessen the usage of makeup and hair dye, in order to avoid contact with chemical exposure. Furthermore, Ketamine has often been linked with IC/BPS in Taiwan. Therefore, we aimed to review whether these IC/BPS patients frequented entertainment venues (ex. night clubs) which could be a possible source of second-hand Ketamine exposure. It has been reported that IC/BPS is often related to smoking ^8–11^, thus, we became interested in investigating whether or not this prompted them to quit smoking. In the Chinese culture, many choose to try Chinese herbs and dietary supplements for ailments^12^, which is a tendency commonly seen in our clinical observation. Therefore, we believe that behavioral change in IC/BPS patients are not limited to food consumption or bathroom habits^12,13^.

In this study, we investigated the lifestyle behavioral changes among patients with IC/BPS and compared them with those of controls without IC/BPS. The results of this study can further understanding, and give insight, into the lifestyle modifications patients make to cope with their IC/BPS symptoms.

## Results

This trial recruited patients from August 2018 to June 2019, with a total of 86 patients in the IC/BPS study group and 86 non-IC/BPS controls. All participants understood the questionnaire and answered all questions independently.

### First part of questionnaire: personal information

The demographic data, clinical symptoms, and medical history of patients with IC/BPS and non-IC/BPS controls are shown in Table 1. With regard to the frequency of urination, frequency of nocturia, and sensation of urinary urgency, patients with IC/BPS were statistically significantly more likely to have these symptoms than the non-IC/BPS controls were (mean daytime urinary frequency, 12.29 ± 5.27 vs. 3.10 ± 3.05; mean nocturia frequency, 2.73 ± 2.22 vs. 0.49 ± 0.61; percentage of participants reporting urinary urgency, 77.91% vs. 5.81%; *P* < 0.0001). Patients with IC/BPS were also statistically significantly more likely to have a family history of IC/BPS than were the non-IC/BPS controls (22.09% vs. 2.33%, *P* < 0.001). The mean duration between onset of symptoms and arrival at our clinic as a result of poor control was 6.04 (mean: 6.04 ± 6.07) years.

**Table 1:** Demographics, clinical symptoms, and medical history of patients with IC/BPS and non-IC/BPS controls.

M ± SD or N (%)	IC/BPS (N = 86)	Non-IC/BPS controls (N = 86)	*P* value
Age (years)	45.81 ± 15.02	44.58 ± 12.66	0.56^a^
Frequency of urination (times/day)	12.29 ± 5.27	3.10 ± 3.05	< 0.001^a^
Nocturia (times/night)	2.73 ± 2.22	0.49 ± 0.61	< 0.001^a^
Urgency	67 (77.91%)	5 (5.81%)	< 0.001^b^
Family history	19 (22.09%)	2 (2.33%)	< 0.001^b^
Allergy history	38 (44.19%)	28 (32.56%)	0.12^b^
Medical disease	29 (33.72%)	17 (19.77%)	0.039^b^
Duration of onset (year)	6.03 ± 6.11	NA	**–**

*M* mean, *N* number, *SD* standard deviation; medical diseases including hypertension, diabetes mellitus, asthma, rheumatoid arthritis, systemic lupus erythematosus, Sjogren’s syndrome, and various renal/hepatic diseases.

^a^*t* test, ^b^Chi-square test.

### Second part of questionnaire: daily environment

A comparison of the air quality and living and workplace environments to which patients and controls reported being typically exposed is shown in Table 2. There was no statistically significant difference in air quality exposure between the groups. Patients in the study group had a statistically lower frequency of having contact with work garments compared with the control group (3.49% vs. 15.12%, *P* < 0.05). Patients with IC/BPS reported more frequent exposure to second-hand smoke than did the non-IC/BPS controls, with the difference being statistically significant (mean: 41.86% vs.18.60%; *P* < 0.05). However, there was no statistically significant difference between the study group and control group with respect to exposure to chemical odors and indoor fragrances in their home living environments (26.74% vs. 17.44%; *P* > 0.05) or in their workplace environments (38.37% vs. 48.84% and 20.93% vs. 16.28%, respectively; *P* > 0.05).

**Table 2:** Comparison of air quality and exposure to living and workplace environments.

	IC/BPS (N = 86)	Non-IC/BPS controls (N = 86)	*P* value
	n (%)	n (%)	
Exposure to chemical odors or fragrances at workplace	18 (20.93%)	14 (16.28%)	0.43^a^
Enclosed space at workplace	33 (38.37%)	42 (48.84%)	0.17^a^
High-risk storage at workplace	7 (8.14%)	2 (2.33%)	0.17^b^
Requirement of wearing special work garments	3 (3.49%)	13 (15.12%)	0.009^a^
Exposure to indoor cooking smoke and unusual smells at home	23 (26.74%)	15 (17.44%)	0.14^a^
Secondhand smoke	36 (41.86%)	16 (18.60%)	< 0.001^c^

*N* number.

^a^Chi-square test, ^b^Fisher’s exact test, ^c^*t* test.

### Third part of questionnaire: consumption of coffee and food and smoking behavior

Comparisons of food consumption habits and frequency (times/month) between the groups are shown in Table 3. There was a statistically significant difference between the patients with IC/BPS and non-IC/BPS controls with regard to the frequency of eating spicy foods (5.70 ± 7.56 vs. 9.47 ± 9.23, *P* < 0.05) and drinking coffee (mean: 9.67 ± 10.64 vs. 16.81 ± 11.37, *P* < 0.001). Patients with IC/BPS also demonstrated a higher frequency of having a smoking habit than the non-IC/BPS controls, with a statistically significant difference (mean: 18.60% vs. 5.81%; *P* < 0.05). However, there was no statistically significant difference between the study group and the control group in eating habits, namely eating barbequed food and eating a plant-based diet (mean: 1.40 ± 1.50 vs. 1.83 ± 3.27 and 3.06 ± 5.95 vs. 3.36 ± 6.36, respectively; *P* > 0.05). Similarly, there was no statistically significant difference between the study group and control group with respect to consuming outside beverages and having a tea-drinking habit (mean: 5.19 ± 6.61 vs. 5.77 ± 6.60 and 7.59 ± 8.48 vs. 8.38 ± 8.56, respectively; *P* > 0.05).

**Table 3:** Comparison of smoking, tea, coffee, and spicy food consumption habits.

	IC/BPS (N = 86)	Non-IC/BPS controls (N = 86)	*P* value
	M ± SD	M ± SD	
Smoking habit, n (%)	16 (18.60%)	5 (5.81%)	0.010^a^
Coffee drinking (times/month)	9.67 ± 10.64	16.81 ± 11.37	< 0.001^b^
Tea drinking (times/month)	7.59 ± 8.48	8.38 ± 8.56	0.54^b^
Takeout beverage consumption (times/month)	5.19 ± 6.61	5.77 ± 6.60	0.56^b^
Barbequed food consumption (times/month)	1.40 ± 1.50	1.83 ± 3.27	0.27^b^
Vegetarian food consumption (times/month)	3.06 ± 5.95	3.36 ± 6.36	0.75^b^
Spicy food consumption (times/month)	5.70 ± 7.56	9.47 ± 9.23	0.004^b^

*M* mean, *SD* standard deviation.

^a^Chi-square test, ^b^*t* test.

### Fourth part of questionnaire: dietary supplements

A comparison between various types of dietary supplements is shown in Table 4. Participants in the study group reported more frequently consuming dietary supplements and traditional Chinese medicine than did those in the control group, but without a statistically significant difference (mean: 10.92 ± 11.38 vs. 10.00 ± 10.60 and 4.37 ± 8.59 vs. 3.01 ± 6.86, respectively; *P* > 0.05). The number of participants who reported eating cranberries in the study group was statistically significantly higher than that in the control group (45.34% vs. 5.81%, *P* < 0.05). However, certain supplement items, except cranberries, exhibited no statistically significant difference between the study group and the control group (vitamins: 38.37% vs. 43.02%, glucosamine/calcium supplement: 22.09% vs. 12.79%, lutein: 23.25% vs. 22.09%, and isoflavone: 3.48% vs. 0.00%; *P* > 0.05).

**Table 4:** Comparison of various types of diary supplements and Chinese herbs.

N (%)	IC/BPS (N = 86)	Non-IC/BPS controls (N = 86)	*P* value
Number	55 (63.95%)	54 (62.79%)	0.87^a^
Frequency of using dietary supplements (times/month)	10.92 ± 11.38	10.00 ± 10.60	0.58^b^
Vitamins	33 (38.37%)	37 (43.02%)	0.53^a^
Glucosamine/calcium	19 (22.09%)	11 (12.79%)	0.11^a^
Lutein	20 (23.25%)	19 (22.09%)	0.86^a^
Isoflavone	3 (3.48%)	0 (0.00%)	0.25^c^
Cranberry	39 (45.34%)	9 (5.81%)	< 0.001^a^
Chinese herbs	4.37 ± 8.59	3.01 ± 6.86	0.25^b^
Others	16 (18.60%)	6 (6.97%)	0.022^a^

^a^Chi-square test, ^b^*t* test, ^c^Fisher’s exact test.

Vitamins include: Vitamin C, Vitamin B complex, Vitamin D, Vitamin E.

### Fifth part of questionnaire: social activities

The frequency with which patients and controls reported using makeup products and engaging in social activities is shown in Table 5. There was a statistically significant difference between the study group and the control group with regard to the frequency of using makeup (mean: 7.71 ± 10.48 vs. 12.31 ± 12.37, respectively; *P* < 0.05). However, there was no statistically significant difference between the groups with respect to the habit of eating out or using lipstick, skincare products, or hair dye products (mean frequency of eating out, 14.40 ± 10.61 vs. 14.27 ± 9.92; using lipstick, 9.33 ± 10.75 vs. 7.98 ± 10.43; using skincare products, 22.85 ± 9.20 vs. 22.83 ± 9.34; and using hair dye products, 2.76 ± 3.11 vs. 2.34 ± 2.81, respectively; *P* > 0.05). Moreover, there was no statistically significant difference between the groups with respect to the habit of going to nightclubs or other entertainment venues that might increase their possibility of exposure to ketamine (mean: 0.64 ± 1.46 vs. 0.48 ± 0.89, respectively; *P* > 0.05).

**Table 5:** Comparison of frequency of using makeup products and social activities.

M ± SD or N (%)	IC/BPS (N = 86)	Non-IC/BPS controls (N = 86)	*P* value
Eating out (times/month)	14.40 ± 10.61	14.27 ± 9.92	0.94^a^
Makeup products use (days/month)	7.71 ± 10.48	12.31 ± 12.37	0.010^a^
Lipstick use (days/month)	9.33 ± 10.75	7.98 ± 10.43	0.41^a^
Skincare products use (days/month)	22.85 ± 9.20	22.83 ± 9.34	0.99^a^
Hair dye use (times/year)	2.76 ± 3.11	2.34 ± 2.81	0.36^a^
Nightclubs or other entertainment venues visits (times/month)	0.64 ± 1.46	0.48 ± 0.89	0.40^a^

*M* mean, *N* number, *SD* standard deviation.

^a^*t* test.

## Discussion

We investigated the lifestyle modifications made by patients with IC/BPS to cope with their symptoms and compared them with the non-IC/BPS controls. Our data revealed that compared with the non-IC/BPS controls, patients with IC/BPS had a statistically significantly higher frequency of urination, nocturia, and urgency. These symptoms might have negative impacts on several aspects of the quality of life of patients with IC/BPS. These patients drank less coffee and consumed less spicy food to avoid potential worsening of their LUTS^12,14,15^. They also chose to avoid working at workplaces requiring one to wear special work garments and used less makeup, either owing to their fewer social activities or because they usually worked from home.

Patients with IC/BPS more frequently reported having a family history of IC/BPS than controls did (up to 10 times), as shown in Table 1. This suggests the important role of genetic background and/or familiar environmental exposure, which is consistent with the results of related studies^16,17^. A study by Warren et al. revealed greater concordance of IC/BPS among monozygotic than among dizygotic twin pairs, suggesting a genetic susceptibility to IC/BPS^17^. A closer biologic relation to a proband was associated with a higher prevalence of the disease^17^. The results of a large population-based study among young twins by Altman et al. support the notion that genetic factors contribute to the occurrence of IC/BPS in women—twin resemblance was greater in monozygotic than in dizygotic twins^5^. The study indicated that twin similarity is suggestive of a genetic component to the etiology of IC/BPS in women. Further, IC/BPS has many features similar to those of autoimmune diseases, which display complicated gene-environment interactions^18–20^.The influence of environmental factors on IC/BPS and how to avoid such influence warrant further investigation. Further, the recruited patients with IC/BPS in this study had an average onset time of 6 years before their diagnosis was confirmed. A study by Driscoll et al. reported that the duration between the onset of urinary symptoms and the diagnosis of IC/BPS is typically close to 5 years^21^. Hence, our results reflect the current situation of these patients; although most patients have attempted to adjust their dietary habits, the changes have failed to alleviate their symptoms.

Even though the study by Altman et al. suspected that some environmental factors may influence the development of IC/BPS in women, they still are not able to find certain environmental factors that are associated with the increase risk of developing IC/BPS^5^. In accordance to Altman’s study, we discovered that there are no significant difference in the air quality of living and workplace environments between patients with IC/BPS and the non-IC/BPS controls shown in Table 2. IC/BPS patients do not relocate their living environment or workplace because of poor air qualities, including chemical odors or fragrances and even secondhand smoke. On the contrary, in our study, we have found that the patients with IC/BPS tend to avoid wearing special work garments at the workplace (e.g., clothes required to work in dust-free rooms, operating rooms, and similar), compared with the non-IC/BPS controls. We suspected that this is due to the consideration that toilets may not be easily available or accessible during work hours, which is a special concern for patients with IC/BPS. However, the small number of patients in our study (3/86, 3.49%) precludes drawing a strong conclusion of why patients avoid wearing special work garments. We think that patients with IC/PBS would still try to choose modification of daily environment, especially when changing living environment or the workplace would be harder than change in type of job. Especially we have no direct proof of environmental factors relevant to IC/PBS. Therefore, the specific environmental factors involved in the progression toward a chronic state of bladder pain should be determined in further studies^5^.

Many articles have identified an association between smoking and urological diseases, including bladder cancer, prostate cancer, erectile dysfunction, benign prostatic hyperplasia, as well as IC/BPS^8,9^. Former and current smoking were both associated with a higher risk of IC/BPS (odds ratio [OR] 1.5, 95%CI 1.18–1.89 and OR 1.49, 95%CI 1.16–1.92, respectively) in a study by Tettamanti et al.^10^. Smoking possibly acts as an irritant through increased production of platelet-activating factor, which contributes to bladder inflammation^9,11^. Although a review of the literature by Mobley et al*.* did not reveal any studies directly regarding cigarette smoking as a risk factor or etiologic agent for IC/BPS, it is otherwise advises patients with IC/BPS to cease tobacco use, as it may aggravate their symptoms^8^. Smoking has been identified as an aggravating factor of symptoms of IC/BPS by the International Urogynecological Association and other associations; thus, smoking cessation has been recommended for patients with IC/BPS^8^. Our data showed that a higher proportion of patients with IC/BPS than non-IC/BPS controls had a smoking habit. This indicates that most patients with IC/BPS have neglected to heed advice regarding the importance of smoking cessation as a lifestyle modification to alleviate their symptoms, despite strong evidence. A study by Breau et al. reported that many patients with IC/BPS reported being unaware of the variety of self-help modification strategies that exist^22^. Urologists and urogynecologists should provide relevant knowledge and education to patients to help them quit their smoking habit^8^.

Previous studies have shown that consuming caffeinated beverages and spicy food may exacerbate the symptoms of IC/BPS^12^. Tea consumption was associated with an increased risk for IC/BPS (OR 1.26, 95%CI 1.02–1.55 for low tea consumption; OR 1.74, 95%CI 1.24–2.44 for high tea consumption), whereas coffee consumption was not a risk factor for IC/BPS (OR 1.1, 95%CI 0.84–1.45) in a study by Tettamanti et al.^10^. Our patients with IC/BPS and controls did not show differences in tea consumption; however, patients with IC/BPS drank less coffee, which may be attributed to cultural differences between our patients and those in other studies. Restricting consumption of coffee and spicy foods is a common strategy in self-care efforts for IC/BPS^12,22^. In addition to consuming less coffee, our patients also consumed less spicy food. This is because consumption of coffee and spicy food exacerbates urgency, a major symptom among patients with IC/BPS. Therefore, they tend to avoid coffee and spicy food as a coping strategy^7^.

In addition to restricting fluid intake and curbing certain types of foods, some patients with IC/BPS consume cranberries and cranberry products^12^. In our study, there was a statistically significant difference in the frequency of using cranberries as a dietary supplement between patients with IC/BPS and the non-IC/BPS controls. Cranberries have been tested for their clinical relevance in many different conditions. Although they have been deemed ineffective for the treatment of urinary tract infections (UTIs)^23^, there is some evidence that cranberry juice may reduce the number of symptomatic UTIs that a person experiences^24^. Thus, although there is no evidence that cranberries have properties that relieve urinary symptoms^13^ and restricted usage has been recommended by certain guidelines^25^, people continue to believe in their efficacy, and they continue to be a popular supplement.

Our patients with IC/BPS used statistically significantly fewer makeup products, which implies they may be unemployed, have chosen to work at home, or be participating in fewer social events. This indirect evidence implies that patients with IC/BPS might choose to stay at home and avoid social activities because of the bothersome toilet availability issue. Thus, IC/BPS may deprive patients of the ability to work full-time—some of them reported being unemployed owing to their bothersome symptoms^26^. A greater impact of bladder symptoms predicted a greater likelihood of not being working currently, not working on several days owing to pain, having missed a number of work days, and working on more days with symptoms^26^. More depressive symptomatology and a greater number of comorbidities predicted reduced work participation. Patients with IC/BPS are more susceptible to personality trait disorders such as depression and anxiety, resulting in more interpersonal problems^27^. As a result of their symptoms, patients are less likely to engage in social activities owing to their reliance on access to the restroom, as implied by the decreased use of makeup. This, in turn, leads to worsened mental health. Thus, psychological issues should not be ignored when devising strategies for caring for patients with IC/BPS. This accords with our previous finding that patients with IC/BPS have more mental illness, anxiety, and depression, as reported in a nationwide population-based study^4^.

Some guidelines have highlighted the importance of patient education in self-care practice and behavior modification as the first-line treatment for patients with IC/BPS^28^. Our study revealed that patients with IC/BPS may have some coping strategies that entail making lifestyle modifications. Hence, it is important for these patients to strengthen their knowledge and education to fully change their lifestyle. A higher frequency of having a family history of IC/BPS was observed in the patients with IC/BPS, but more detailed research in pathophysiology and etiology should be constructed. With more understanding of gene-environmental etiology, this would help tremendously with future lifestyle modification Our study offers a preliminary result owing to the relatively small number of participants. Larger scale studies with more participants are warranted to investigate the feasibility and effectiveness of this regimen before drawing definite conclusions.

## Methods

A prospective study was conducted between August 2018 and June 2019. Data collection was approved by the Taipei Medical University-Joint Institutional Review Board (No: 201807093), Taipei, Taiwan. We obtained informed consent from all patients by themselves before they were enrolled. All experiments were performed in accordance with relevant named guidelines and regulations. Our questionnaire examines the difference between the groups of healthy participants and IC/BPS group in diet, toilet habits, choice of living and workplace, usage of makeup and hair-dye, usage of Chinese herbal medication and dietary supplements based on the concepts of various articles^5,7,10,12,13, 26,29^.

### Inclusion and exclusion criteria

IC/BPS was defined as “an unpleasant sensation (pain, pressure, discomfort) perceived to be related to the urinary bladder, associated with LUTS for more than six weeks duration, in the absence of infection or other identifiable causes” by the Society for Urodynamics and Female Urology in 2009^30^. According to the European Society for the Study of Interstitial Cystitis, BPS is diagnosed according to the presence of chronic pelvic pain or discomfort related to the bladder and at least one other LUTS, such as urinary frequency or urgency^2^. The criteria for enrollment of female patients (from our gynecological outpatient department) with newly diagnosed with IC/BPS in our study were the typical diagnostic symptoms and findings from cystoscope examination. The characteristic presentation of IC/BPS includes suprapubic pain with a full bladder with frequency, nocturia, and urgency for at least 6 weeks^31^. Further, pelvic pain in patients with IC/BPS generally worsens with a full bladder and improves with urination^32–34^. These diagnostic symptoms were the patients’ chief complaints for more than 6 weeks and had serious consequences on their quality of life. A 3-day urinary diary was used to quantify the effects of these symptoms, and record urgency that has interfered with quality of life or prompted to seek for medical help. Cystoscopic examination was performed to confirm IC/BPS in patients with glomerulations or Hunner’s ulcers on their bladder wall. Patients who were clinically symptomatic with Hunner’s ulcers or those in whom glomerulations were observed through cystoscopy were included.

The age-matched non-IC/BPS controls were selected from among the colleagues of the clinicians, including nurses, administrative staff, and medical students, who had no symptoms of OAB or IC/BPS. According to IRB regulations, all participants were older 20 years of age. All individuals were literate, and capable of understanding our questionnaire; also, there simultaneously was a clinical assistant to annotate in detail.

Patients with direct exposure to ketamine, second hand exposure ketamine, or those with unconfirmed cystoscopic findings were excluded. In addition, those who refused to answer the questionnaire; those aged below 20 years; and those with related pathological conditions such as upper or lower UTIs, urethral diverticulum, urogenital tract malignancy, a history of urinary tract stones, or presence of a pelvic mass or malignancy were excluded. Further, patients with a markedly enlarged uterus, as determined through sonographic examination, were also excluded.

### Contents of questionnaire

All participants were asked to complete our self-developed questionnaire, which consisted of five sections. The first part collected each patient’s personal information, such as frequency of nocturia, allergies, a family history of IC/BPS, and a history of medical diseases (including hypertension, diabetes mellitus, asthma, rheumatoid arthritis, systemic lupus erythematosus, Sjogren’s syndrome, and various renal/hepatic diseases); and participants with medical diseases were all under good control with medication treatment. The second part collected information concerning the patient’s living environment, work environment, air conditions in these environments, skin contact with work garments, and secondhand smoke inhalation. The third part solicited information regarding the patient’s daily life habits. With respect to food, the information collected concerned the frequency of consuming takeout beverages and whether the patient had a preference for barbequed food, spicy food, or vegetarian food. Smoking habits were a topic of emphasis in this section. The fourth section concerned the frequency of using dietary supplements and their various types. The last section concerned daily habits, with emphasis on social activities, including eating out, the use of makeup or hair dye, and contact with the environment at entertainment venues.

### Statistical analysis

The Statistical Package for Social Sciences (SPSS v.22) was used for data management and statistical analysis. Continuous variables are presented as averages and standard deviation (SD). Categorical variables are presented as counts and percentages. Continuous variables between patients with IC/BPS and non-IC/BPS controls were compared using the independent sample *t* test, and the categorical variables were compared using the chi-square test. A *P* value < 0.05 indicated statistical significance throughout this study.

### Ethical approval

The material contained in the manuscript has not been previously published and is not being concurrently submitted elsewhere.

## Data Availability

The datasets generated during and/or analysed during the current study are available from the corresponding author on reasonable request.
